# The Protein Precursors of Peptides That Affect the Mechanics of Connective Tissue and/or Muscle in the Echinoderm *Apostichopus japonicus*

**DOI:** 10.1371/journal.pone.0044492

**Published:** 2012-08-31

**Authors:** Maurice R. Elphick

**Affiliations:** Queen Mary University of London, School of Biological and Chemical Sciences, London, United Kingdom; Washington University Medical School, United States of America

## Abstract

Peptides that cause muscle relaxation or contraction or that modulate electrically-induced muscle contraction have been discovered in the sea cucumber *Apostichopus japonicus* (Phylum Echinodermata; Class Holothuroidea). By analysing transcriptome sequence data, here the protein precursors of six of these myoactive peptides (the SALMFamides Sticho-MFamide-1 and -2, NGIWYamide, stichopin, GN-19 and GLRFA) have been identified, providing novel insights on neuropeptide and endocrine-type signalling systems in echinoderms. The *A. japonicus* SALMFamide precursor comprises eight putative neuropeptides including both L-type and F-type SALMFamides, which contrasts with previous findings from the sea urchin *Strongylocentrotus purpuratus* where L-type and F-type SALMFamides are encoded by different genes. The NGIWYamide precursor contains five copies of NGIWYamide but, unlike other NG peptide-type neuropeptide precursors in deuterostomian invertebrates, the NGIWYamide precursor does not have a C-terminal neurophysin domain, indicating loss of this character in holothurians. NGIWYamide was originally discovered as a muscle contractant, but it also causes stiffening of mutable connective tissue in the body wall of *A. japonicus*, whilst holokinins (PLGYMFR and derivative peptides) cause softening of the body wall. However, the mechanisms by which these peptides affect the stiffness of body wall connective tissue are unknown. Interestingly, analysis of the *A. japonicus* transcriptome reveals that the only protein containing the holokinin sequence PLGYMFR is an alpha-5 type collagen. This suggests that proteolysis of collagen may generate peptides (holokinins) that affect body wall stiffness in sea cucumbers, providing a novel perspective on mechanisms of mutable connective tissue in echinoderms.

## Introduction

Peptides are evolutionarily ancient mediators of intercellular communication. In the animal kingdom, the use of secreted peptides as intercellular signalling molecules has probably been exploited most extensively in nervous systems. Thus, neurons release “neuropeptide” signalling molecules, which act as neurotransmitters, neuromodulators or neurohormones and have important roles in co-ordination of physiological processes and whole-animal behaviour [1]. Neuropeptides are derived from larger precursor proteins, a key character of which is an N-terminal signal peptide that targets them to the lumen of the endoplasmic reticulum as the first step towards the regulated secretory pathway. Neuropeptides are cleaved from their precursor protein by endopeptidases that target monobasic or dibasic sites. In some cases a single bioactive neuropeptide molecule is derived from each neuropeptide precursor protein. However, perhaps more typically multiple copies of neuropeptides are derived from each precursor protein, either as multiple identical copies or as a variety of structurally related forms.

Research on neuropeptide signalling systems has, of course, largely focused on mammals and other vertebrates. However, early on it was recognised that invertebrates are attractive as model systems for neuropeptide studies because of the relative simplicity of their nervous systems [2], [3]. Furthermore, an understanding of how neuropeptide signalling systems have evolved requires analysis of species from a variety of animal phyla [4].

Echinoderms (e.g. sea cucumbers, sea urchins, starfish) are of particular interest for phylogenetic studies because they are one of only three non-chordate phyla that are deuterostomes [5]. Furthermore, many aspects of the biology of echinoderms are fascinating from a physiological perspective. For example, echinoderms have “mutable connective tissue”. Thus, the stiffness of collagenous tissue in echinoderms can change rapidly and there is evidence that this is controlled by the nervous system [6]. The underlying molecular and cellular mechanisms of mutable connective tissue are not understood, but there is evidence that neuropeptides mediate neural control of connective tissue stiffness in echinoderms [7].

Research on neuropeptides in echinoderms is still in its infancy, but opportunities for rapid progress are being provided by sequencing of the genomes and/or transcriptomes of echinoderm species [8], [9], [10]. The first paper to report the identification of neuropeptides in an echinoderm was published in 1991 [11]. Thus, the SALMFamide neuropeptides S1 (GFNSALMFamide) and S2 (SGPYSFNSGLTFamide) were purified from radial nerve extracts of the starfish species *Asterias rubens* and *Asterias forbesi* on account of their immunoreactivity with antibodies to a molluscan FMRFamide-related neuropeptide pQDPFLRFamide. Subsequently, two SALMFamide neuropeptides (GFSKLYFamide and SGYSVLYFamide) were isolated from another echinoderm species, the sea cucumber *Holothuria glaberrima* [12]. Hence, the concept of a SALMFamide neuropeptide family in the echinoderms emerged, with the C-terminal sequence SxLxFamide apparently a characteristic motif. Immunocytochemical studies and *in vitro* pharmacological studies have revealed that SALMFamide neuropeptides are widely expressed in echinoderm nervous systems and act as inhibitory neurotransmitters, causing relaxation of a variety of different echinoderm muscle preparations [13], [14], [15], [16], [17], [18], [19], [20], [21].

An alternative functional-based strategy for identification of echinoderm neuropeptides was used by Iwakoshi et al. to screen extracts of the sea cucumber *Apostichopus japonicus* for peptides that affect the *in vitro* contractility of the radial longitudinal muscle (RLM) from this species [22]. Contracting and relaxing actions as well as modulatory effects on electrically induced contractions were assayed and fifteen peptides that affect the contractile activity of the RLM were identified. Subsequently, Ohtani et al. (1999) extended the analysis of myoactive peptides in *A. japonicus* by incorporating the use of intestine preparations as a bioassay system alongside the RLM [23]. An additional five peptides were identified, increasing the number of putative neuropeptides identified to twenty and the names, amino acid sequences and bioactivities of these peptides are shown in table 1.

**Table 1:** Bioactive peptides isolated from the body wall of the sea cucumber *Apostichopus japonicus*.

Peptide name	Peptide sequence	Effect on RLM	Effect on intestine	Effect on body wall
Sticho-MFamide-1	GYSPFMFamide	No effect	R	-
Sticho-MFamide-2	FKSPFMFamide	No effect	R	-
NGIWYamide	NGIWYamide	C	C	stiffening
Stichopin	DRQGWPACYDSKGNYKC	I	No effect	IA
GN-19	GGRLPNYAGPPRMPWLIHN	No effect	C or R	-
GLRFA	GLRFA	P	C	-
Holokinin-1	PLGYMFR	I	C	softening
Holokinin-2	PLGYM(O)FR	I	C	softening
Holokinin-3	PLGY(Br)M(O)FR	I	C	-
Holokinin-1 (3–7)	GYMFR	I	C	-
SWYG-1	SWYGSLG	I or P	No effect	-
SWYG-2	SWYGTLG	I or P	No effect	-
SWYG-3	SWYGSLA	I	No effect	-
-	GYI	I	-	-
KIamide-9	KHKTAYTGIamide	I	C	-
-	GYWKDLDNYVKAHKT	I	-	-
-	MPMNPADYFSRGTVYIPTRDS	P	-	-
-	GPSANSFTYGRCADDRC	P	-	-
-	KGKQKGAGRGKGN	P	-	-
-	APHAIRPPSG	I	-	-

Sequences of twenty peptides isolated from the body wall of the sea cucumber *Apostichopus japonicus* that affect the contractile activity of the radial longitudinal muscle (RLM) and/or the intestine. Four of the peptides also affect the stiffness of the collagenous body wall. Key: I, inhibition of electrically-induced contraction; P, potentiation of electrically-induced contraction; C, contraction; R, relaxation.; IA, inhibition of acetylcholine-induced stiffening. (Iwakoshi et al., 1995; Birenheide et al., 1998; Ohtani et al., 1999).

Amongst the peptides identified by Ohtani et al. (1999) were two peptides (GYSPFMFamide or “Sticho-MFamide-1” and FKSPFMFamide or “Sticho-MFamide-2”) that cause relaxation of the intestine in *A. japonicus*. Analysis of the sequences of these peptides revealed that they are structurally similar to the SALMFamide neuropeptides previously identified in the sea cucumber *H. glaberrima* (GFSKLYFamide and SGYSVLYFamide). However, the *A. japonicus* peptides have a phenylalanine (F) residue in the position occupied by a leucine (L) residue in the *H. glaberrima* peptides, broadening the structural characteristics of SALMFamide neuropeptides to SxF/LxFamide [14].

In addition to the SALMFamide peptides that cause relaxation of the *A. japonicus* intestine, a peptide that causes contraction of both the intestine and the RLM was identified as NGIWYamide [22], [23]. Subsequently, more detailed investigation of the expression this peptide has revealed that it is expressed by neurons in the radial nerve cords and circumoral nerve ring of *A. japonicus* and in nerve fibres innervating the tube feet, tentacles, intestine and body wall dermis [24]. This pattern of expression is consistent with the notion that NGIWYamide acts as a neuropeptide in sea cucumbers and accordingly it was found that, in addition to its contractile effect on the RLM [22], NGIWYamide also causes contraction of tentacle preparations and inhibition of the rhythmic contractile activity of intestine preparations [24].

Interestingly, four of the peptides originally identified in *A. japonicus* as myoactive peptides also affect the stiffness of collagenous body wall preparations from this species. Thus, NGIWYamide causes stiffening of the body wall, whereas holokinin-1 and holokinin-2 both cause softening of the body wall. The peptide stichopin, which inhibits electrically induced contractions of the RLM, also exerts an inhibitory effect on body wall preparations, blocking acetylcholine induced stiffening of the body wall. Accordingly, it was proposed that these peptides act *in vivo* as neuropeptides that control the stiffness of connective tissue in the body wall [7]. Consistent with this notion, immunocytochemical studies have revealed that NGIWYamide-immunoreactivity and stichopin-immunoreactivity are present in nerve fibres that project into the connective tissue layer of the body wall [24], [25]. However, it is not known how NGIWYamide and stichopin exert effects on the body wall dermis. Furthermore, it is also not known if holokinin-1 and holokinin-2, which cause softening of the dermis *in vitro*, are also neuropeptides that are released by nerve fibres innervating the body wall.

Whilst there is immunocytochemical evidence that both NGIWYamide and stichopin are realeased by neurons and therefore act as neuropeptide signalling molecules in *A. japonicus*, there is at present no such evidence available for any of the other eighteen myoactive peptides shown in table 1. Further insights on this issue could be obtained if the protein precursors from which these peptides are derived were identified. Opportunities to do this have been provided recently by sequencing of the transcriptome of *A. japonicus*, with identification of 29,666 transcripts (isotigs) [10]. Here I have analysed this dataset in an effort to identify the protein precursors of the twenty myoactive peptides from *A. japonicus* that are shown in table 1. Protein precursors for ten of the twenty myoactive peptides have been identified, providing new insights on the molecular biology of peptide signalling systems and their roles as regulators of connective tissue and/or muscle in echinoderms.

## Materials and Methods

A file containing the sequences of 29,666 *A. japonicus* isotigs in FASTA format was downloaded from the supporting information section (Dataset S1) of the online paper by Du et al. [10], which is available from the PloSONE website (http://www.plosone.org/article/info%3Adoi%2F10.1371%2Fjournal.pone.0033311). The data was then subject to BLAST analysis [26], as described below, using SequenceServer software (http://www.sequenceserver.com/), which is freely available to academic users [27].

To search for transcripts encoding putative precursor proteins for the myoactive peptides listed in table 1, the sequences of these peptides were submitted individually as queries in tBLASTn searches of the isotig database (29,666 isotigs), with the BLAST parameter e-value set to 1000 [26]. The sequences were submitted as just the peptide sequence alone and/or as multiple copies of each peptide sequence in tandem, separated by putative dibasic cleavage sites (Lys-Arg). Isotigs identified as encoding putative precursors were analysed after translation of their full length DNA sequence into protein sequence using the ExPASy Translate tool (http://web.expasy.org/translate). Proteins were assessed as potential precursors of secreted bioactive peptides by investigating a). the presence of putative monobasic or dibasic cleavage sites on the N-terminal and C-terminal side of the sequence of the bioactive peptide and b). the presence of a putative N-terminal signal peptide sequence, using the SignalP 3.0 server at http://www.cbs.dtu.dk/services/SignalP-3.0/ [28].

For some of the isotigs identified as cDNAs enoding putative precursor proteins it was necessary to perform additional analysis of raw sequence data, which can be downloaded from the NCBI Short Read Archive (SRA) database (http://www.ncbi.nlm.nih.gov/sra/) under the accession number SRA046386.

## Results

### Identification of a SALMFamide neuropeptide precursor protein

**Figure 1:**
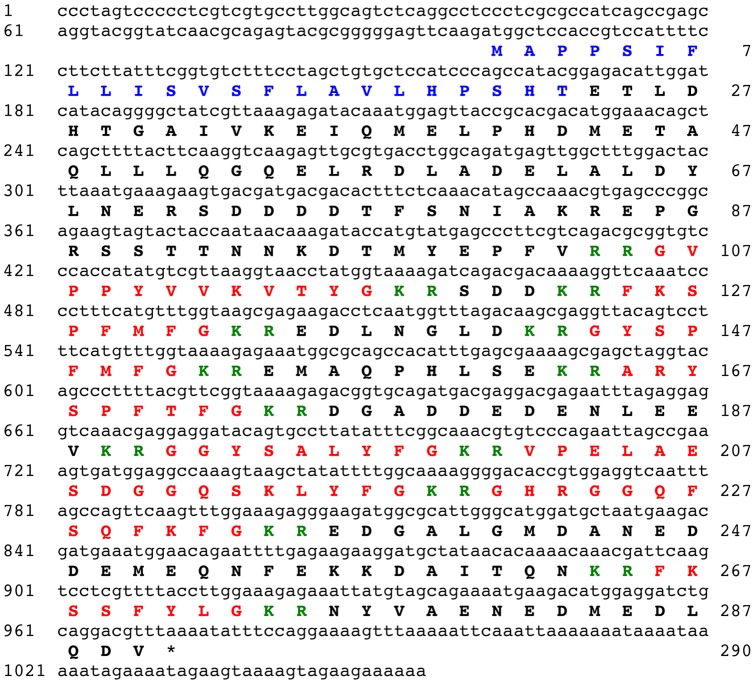
The *Apostichopus japonicus* SALMFamide precursor. The cDNA sequence (lowercase, 1054 bases) encoding the SALMFamide precursor protein (bold uppercase, 290 amino acid residues) is shown. The predicted signal peptide is shown in blue and the eight putative SALMFamide neuropeptides are shown in red, flanked by putative dibasic cleavage sites (KR or RR) shown in green. The asterisk shows the position of the stop codon.

BLAST analysis of *A. japonicus* isotigs using a query comprising sequences of the two F-type SALMFamides previously identified in this species bounded by putative dibasic cleavage sites (KRGYSPFMFGKRFKSPFMFGKR) resulted in the identification of isotig 12925 as a putative SALMFamide precursor cDNA. However, translation of the 900 base sequence of isotig 12925 revealed that the FKSPFMFG and GYSPFMFG sequences were encoded on different reading frames. Therefore, raw sequence data was analysed and assembled manually to identify a full-length open reading frame encoding a 290-residue precursor protein, which comprises an N-terminal signal peptide and eight putative amidated neuropeptides bounded by dibasic cleavage sites (Figure 1). These include GYSPFMFamide (Sticho-MFamide-1) and FKSPFMFamide (Sticho-MFamide-2) and three other putative F-type SALMFamide neuropeptides: ARYSPFTFamide, GHRGGQFSQFKFamide and FKSSFYLamide. The precursor also contains two putative L-type SALMFamide neuropeptides, GGYSALYFamide and VPELAESDGGQSKLYFamide, and the putative peptide GVPPYVVKVTYamide.

### Identification of the NGIWYamide precursor protein

**Figure 2:**
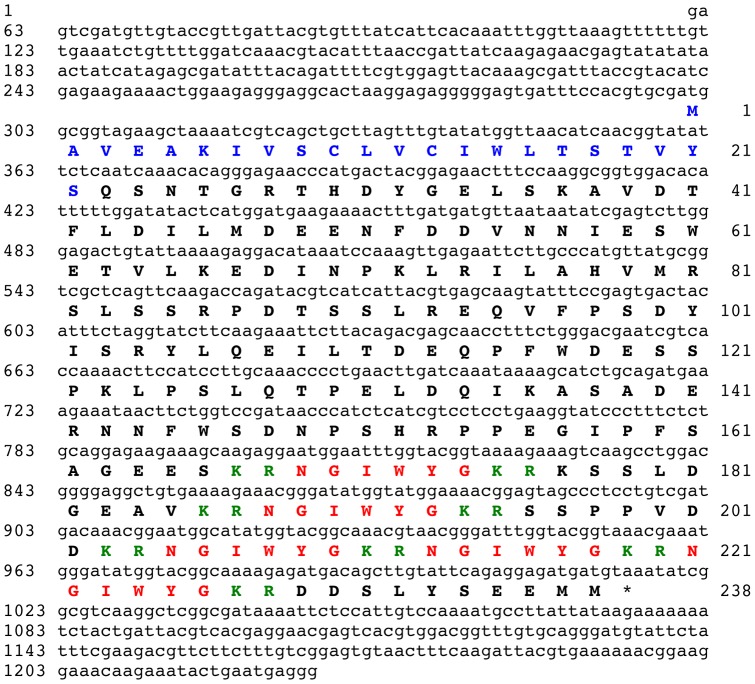
The *Apostichopus japonicus* NGIWYamide precursor. The cDNA sequence (lowercase, 1226 bases) encoding the NGIWYamide precursor protein (bold uppercase, 238 amino acid residues) is shown. The predicted signal peptide is shown in blue and the five copies of NGIWYamide are shown in red, flanked by putative dibasic cleavage sites (KR) shown in green. The asterisk shows the position of the stop codon.

BLAST analysis of the *A. japonicus* isotigs using a query comprising three copies of the sequence NGIWYG bounded by putative dibasic cleavage sites resulted in the identification of isotig 16278 (1226 bases) as a cDNA encoding the NGIWYamide precursor. The 238-residue NGIWYamide precursor comprises an N-terminal signal peptide and five copies of the sequence NGIWYG, which are located in the C-terminal half of the protein from residues 169 to 226 (Figure 2).

### Identification of the stichopin precursor protein

**Figure 3:**
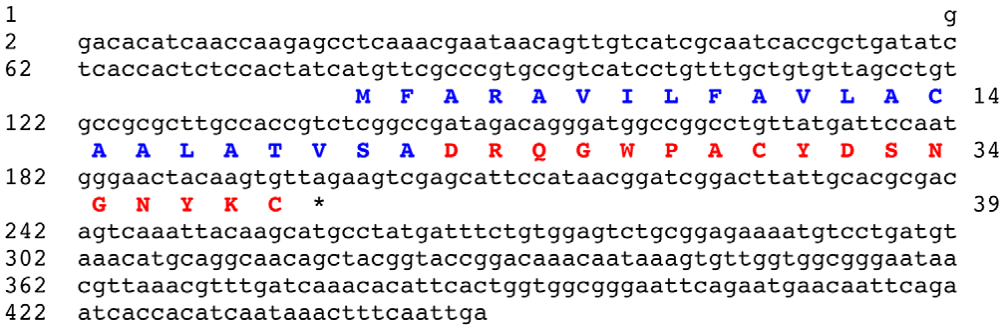
The *Apostichopus japonicus* stichopin precursor. The cDNA sequence (lowercase, 449 bases) encoding the stichopin precursor protein (bold uppercase, 39 amino acid residues) is shown. The predicted signal peptide is shown in blue and the stichopin peptide sequence is shown in red. The asterisk shows the position of the stop codon.

BLAST analysis of the *A. japonicus* isotigs using a query comprising the sequence of stichopin (DRQGWPACYDSKGNYLC) resulted in the identification of isotig 26630 (449 bases) as a cDNA encoding the stichopin precursor. Isotig 26630 contains an open reading frame encoding a 39 residue protein comprising an N-terminal signal peptide (residues 1–22) followed by the 17 residue sequence DRQGWPACYDSNGNYKC, which is identical to stichopin in all but two amino acid residues (underlined; residues 12 and 16) (Figure 3). Analysis of raw sequence data revealed a polymorphism in the codon for residue 12, with a codon for an asparagine residue (N) found in 9 out of 16 reads (consistent with the sequence of isotig 26630) and a codon for a lysine residue (K) found in 7 out of 16 reads (consistent with the published sequence of the stichopin peptide). However, analysis of raw sequence data revealed that a codon for a lysine residue at position 16 was found in all 16 reads.

### Identification of the GN-19 precursor protein

**Figure 4:**
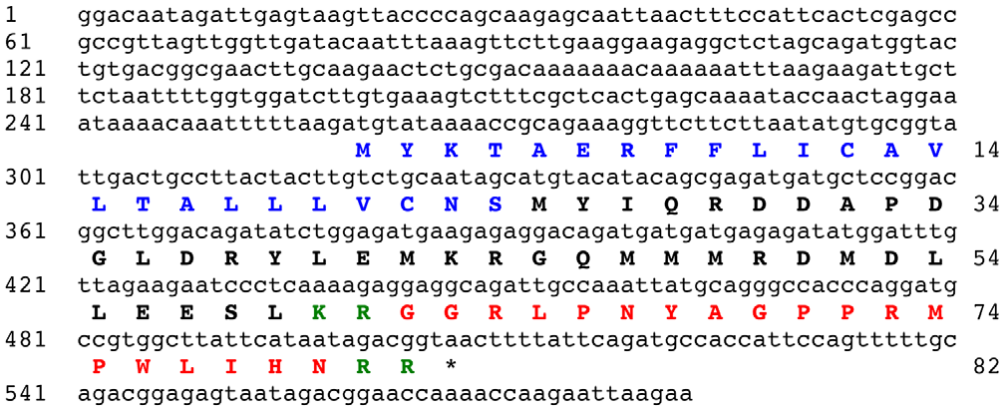
The *Apostichopus japonicus* GN-19 precursor. The cDNA sequence (lowercase, 582 bases) encoding the GN-19 precursor protein (bold uppercase, 82 amino acid residues) is shown. The predicted signal peptide is shown in blue and the GN-19 peptide sequence is shown in red, flanked by putative dibasic cleavage sites (KR, RR) shown in green. The asterisk shows the position of the stop codon.

BLAST analysis of the *A. japonicus* isotigs using a query comprising the sequence of GN-19 (GGRLPNYAGPPRMPWLIHN) resulted in the identification of isotig 23619 (582 bases) as a cDNA encoding the GN-19 precursor. Isotig 23619 contains an open reading frame encoding a 82 residue protein comprising an N-terminal signal peptide (residues 1–24) and the GN-19 peptide sequence, which is located from residues 52 to 80 bounded by putative dibasic cleavage sites (Figure 4).

### Identification of the GLRFA precursor protein

**Figure 5:**
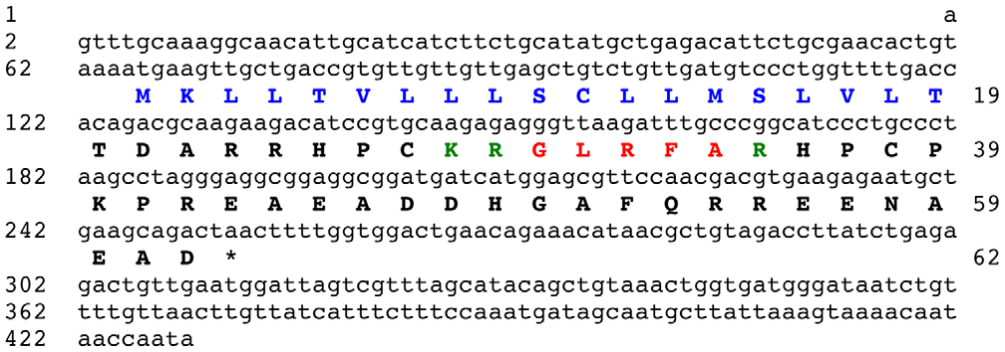
The *Apostichopus japonicus* GLRFA precursor. The cDNA sequence (lowercase, 429 bases) encoding the GLRFA precursor protein (bold uppercase, 62 amino acid residues) is shown. The predicted signal peptide is shown in blue and the single copy of the GLRFA peptide sequence is shown in red, flanked N-terminally by a putative dibasic cleavage site (KR) and C-terminally by a putative monobasic cleavage site (R), which are shown in green. The asterisk shows the position of the stop codon.

BLAST analysis of the *A. japonicus* isotigs using a query comprising the GLRFA sequence bounded by putative dibasic cleavage sites (KRGLRFAKR) resulted in the identification of isotig 12170 (429 bases) as a cDNA encoding the GLRFA precursor. Isotig 12170 contains an open reading frame encoding a 62 residue protein comprising an N-terminal signal peptide (residues 1–19) and the GLRFA peptide sequence, which is located from residues 30 to 34 bounded N-terminally by a putative dibasic cleavage site and bounded C-terminally by a putative monobasic cleavage site (Figure 5).

### Identification of a partial protein sequence containing the holokinin sequence PLGYMFR

**Figure 6:**
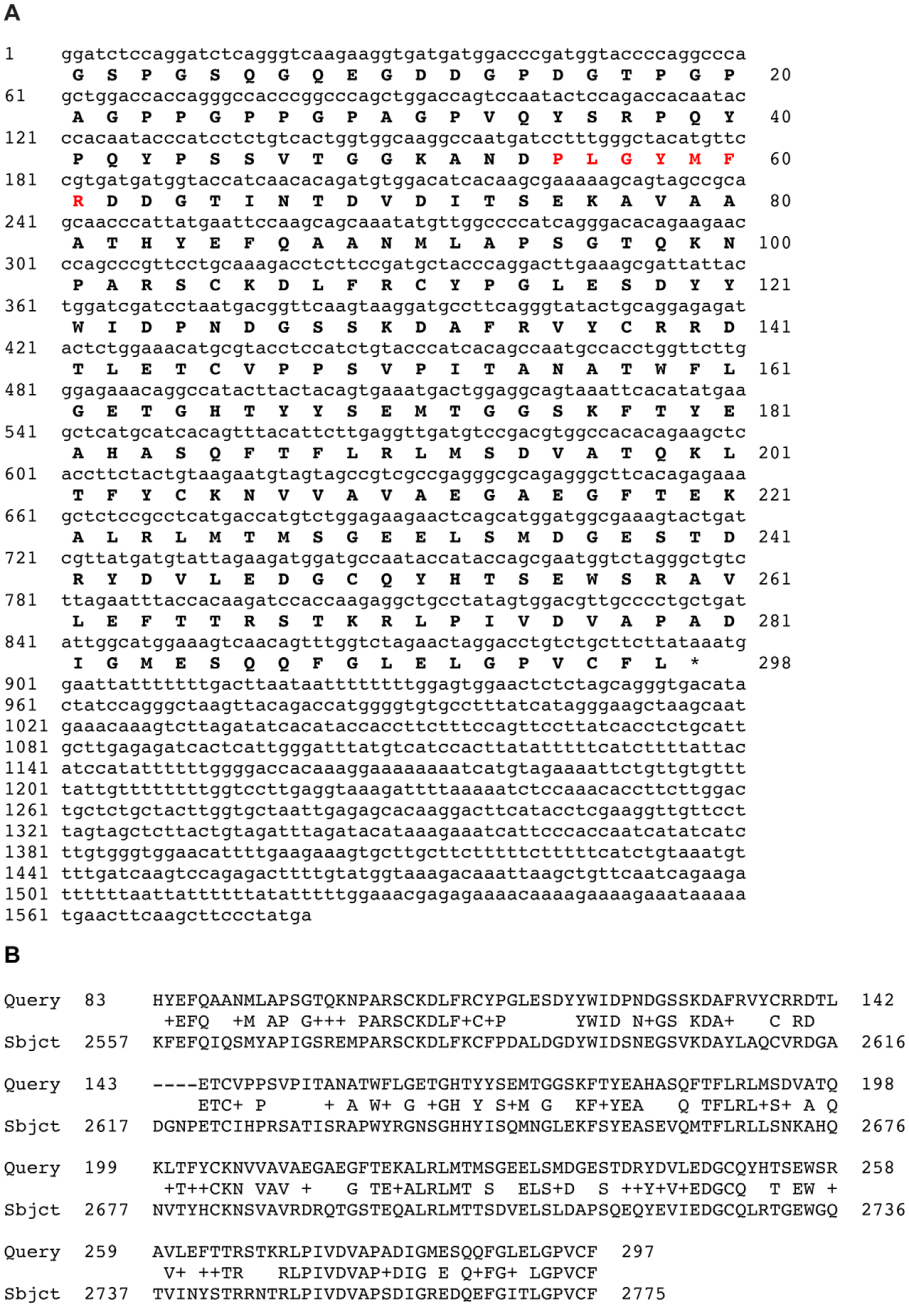
The holokinin peptide sequence PLGYMFR is present in *Apostichopus japonicus* alpha-5 type collagen. **A.** The sequence of isotig 14428 is shown (lowercase, 1582 bases) with the partial sequence of the protein that it encodes shown underneath in bold uppercase (298 amino acid residues). The holokinin sequence PLGYMFR is shown in red and the asterisk shows the position of the stop codon. **B.** BLAST alignment showing that the protein encoded by isotig 14428 (Query) shares a high level of sequence similarity with the C-terminal region of alpha-5 type collagen from the sea urchin *Strongylocentrotus purpuratus* (Subjct; XP_780851.3, GI:390347052).

BLAST analysis of the *A. japonicus* isotigs using a query comprising the holokinin sequence PLGYMFR resulted in the identification of isotig 14428 (1582 bases) as a partial cDNA encoding a protein sequence comprising 298 residues, with the sequence PLGYMFR located between residues 35 and 41 (Figure 6A). However, the PLGYMFR sequence is not bounded by putative monobasic or dibasic cleavage sites and the protein lacks a putative N-terminal signal peptide sequence. Furthermore, submission of the 298-residue sequence as a query for BLAST analysis of the GenBank protein database revealed that it shares a high level of sequence identity (53% in the region from residues 83–297) with the C-terminal region of alpha-5 type collagen from the sea urchin *Strongylocentrotus purpuratus* (Figure 6B).

### Identification of a partial protein sequence containing the peptide sequence SWYGSLA

BLAST analysis of the *A. japonicus* isotigs using queries comprising the sequences of the peptides SWYG-1 (SWYGSLG) and SWYG-2 (SWYGTLG) did not reveal any transcripts encoding proteins containing these sequences. However, a transcript (isotig 11737) encoding a protein containing the sequence of SWYG-3 (SWYGSLA) was identified. Analysis of isotig 11737 revealed that it contains an open reading frame encoding 137 residues with the sequence SWYGSLA located from residues 125 to 131 (Figure S1). A putative monobasic cleavage site is located at residue 124 but there is not a potential monobasic or dibasic cleavage site on the C-terminal side of the SWYGSLA sequence, which is atypical of precursor proteins for secreted peptide signalling molecules. Furthermore, analysis of the 137-residue sequence did not reveal the presence of a putative N-terminal signal peptide. Interestingly, submission of the 137-residue sequence as a query in a BLAST search of the GenBank protein database revealed sequence similarity with proteins from i). a hyperthermophilic bacterium *Aquifex aeolicus* (NP_214225.1 GI:15606845), ii). a curvibacterial putative symbiont of the cnidarian *Hydra magnipapillata* (CBA26623.1 GI:260219765), iii) the bacterium *Hydrogenivirga* sp. 128-5-R1-1 (ZP_02177525.1 GI:163782528), iv). the bacterium *Prevotella* sp. oral taxon 472 str. F0295 (ZP_05918427.1 GI:260911861) and v). the bacterium *Hydrogenobaculum* sp. Y04AAS1 (YP_002121272.1 GI:195952982). Therefore, the 137-residue protein containing the sequence SWYGSLA may in fact be derived from a bacterial species associated with *A. japonicus*.

### Identification of proteins containing the peptide sequence GYI

BLAST analysis of the *A. japonicus* isotigs using a query comprising the GYI sequence bounded by putative dibasic cleavage sites (KRGYIKR) revealed two isotigs encoding potential precursor proteins for this peptide: isotigs 09011 and 09012. Analysis of the sequences of these isotigs revealed that they both encode the same protein, with a single copy of the sequence GYI bounded by a lysine residue N-terminally and the sequence KRR C-terminally. Furthermore, submission the sequence of the protein encoded by isotigs 09011 and 09012 as a query in a BLAST analysis of the GenBank protein database revealed that it is a sea cucumber homolog of human UFM1-specific ligase 1 (Figure S2). Therefore, the GYI peptide may be derived from UFM1-specific ligase 1 in *A. japonicus* as a consequence of *in vitro* proteolysis of this enzyme. It seems unlikely, however, that this is physiologically relevant as a mechanism of peptide signalling *in vivo.*

### Bioactive peptides from *A. japonicus* for which no putative precursor proteins were identified

BLAST analysis of the *A. japonicus* isotigs with the sequences of the six other myoactive peptides (KHKTAYTGIamide, APHAIRPPSG, GYWKDLDNYVKAHKT, MPMNPADYFSRGTVYIPTRDS, GPSANSFTYGRCADDRC and KGKQKGAGRGKGN) did not reveal any isotigs encoding proteins contain these sequences.

## Discussion

Twenty peptides that affect the mechanical activity of connective tissue and/or muscle have been identified in the sea cucumber *A. japonicus* [7], [22], [23]. However, the physiological relevance of the *in vitro* effects of these peptides on connective tissue and/or muscle is largely unknown. Here this issue was addressed by investigating the identity and properties of the proteins that these peptides are derived from. Six of the peptides (Sticho-MFamide-1 and -2, NGIWYamide, stichopin, GN-19 and GLRFA) are derived from proteins that have an N-terminal signal peptide, indicating that these peptides are secreted as neuropeptide and/or endocrine-type signalling molecules. Furthermore, holokinins (PLGYMFR and related peptides), peptides that cause softening of the body wall in *A. japonicus*, appear to be derived from proteolysis of collagen, which provides an important new insight on the mechanisms of mutable connective tissue in sea cucumbers and possibly other echinoderms.

### The *A. japonicus* SALMFamide precursor is a precursor for both L-type and F-type SALMFamide neuropeptides

GYSPFMFamide (Sticho-MFamide-1) and FKSPFMFamide (Sticho-MFamide-2) were originally discovered as peptides that cause relaxation of the intestine in *A. japonicus* [14], [23] and here the precursor protein for these two F-type SALMFamide peptides has been identified. Consistent with the notion that these two peptides are secreted neuropeptide signalling molecules, the precursor has an N-terminal signal peptide sequence. Furthermore, the precursor protein also contains six other putative neuropeptides. Three of these are also putative F-type SALMFamide neuropeptides: ARYSPFTFamide, GHRGGQFSQFKFamide and FKSSFYLamide. However, the third peptide, FKSSFYLamide, is unusual because it has a C-terminal leucine residue, whereas all of the SALMFamide neuropeptides previously identified in echinoderms have a C-terminal phenylalanine residue [14], [29]. In addition, the precursor contains the putative peptide GVPPYVVKVTYamide, which exhibits even greater structural divergence from the SxL/FxFamide motif that characterises SALMFamides. Indeed, if this peptide were not part of a SALMFamide neuropeptide precursor it is unlikely that it would be recognised as being related to SALMFamides.

Perhaps the most interesting feature of the *A. japonicus* SALMFamide precursor, however, is that in addition to containing F-type SALMFamides, it also contains two putative L-type SALMFamide neuropeptides, GGYSALYFamide and VPELAESDGGQSKLYFamide, which appear to be homologs of the two L-type SALMFamides (SGYSVLYFamide and GFSKLYFamide, respectively) that were originally discovered in the *H. glaberrima* [12], [17], [20]. This is interesting because analysis of sequence data from the sea urchin *S. purpuratus* revealed that in this species F-type and L-type SALMFamide neuropeptides are encoded by different genes, with seven F-type SALMFamides (SpurS1 – SpurS7) derived from one precursor protein and two L-type SALMFamides (SpurS8 and SpurS9) derived from a second precursor protein [29], [30]. The existence of a SALMFamide precursor in *A. japonicus* containing both F-type and L-type SALMFamides may reflect conservation of a characteristic of the common ancestor of sea cucumbers and sea urchins. Accordingly, the existence of separate precursors for F-type and L-type SALMFamides in *S. purpuratus* is presumably a consequence of duplication of a single SALMFamide gene in an ancestor of sea urchins, with subsequent loss of L-type and F-type SALMFamides in the gene copies that gave rise to genes encoding F-type and L-type SALMFamide precursors in *S. purpuratus*, respectively. Thus far, F-type SALMFamides have only been found in sea cucumbers and sea urchins, whereas L-type SALMFamides have been found in all echinoderms for which peptide sequence data are available (starfish, sea cucumbers and sea urchins) [29]. It is possible, therefore, that L-type SALMFamides occur throughout the echinoderms, with F-type SALMFamides being restricted to the echinoderm clade comprising extant holothurians and echinoids. Further insights on this issue will be obtained when genome/transcriptome sequence data are available for species from other extant echinoderm classes (asteroids, ophiuroids and crinoids).

### The NGIWYamide precursor: an NG peptide precursor without a neurophysin domain

NGIWYamide is a pleiotropic neuropeptide because although it was originally discovered on account of its myoactivity [22], [23], more recently NGIWYamide (or “cubifrin”) was also identified as potent inducer of oocyte maturation and gamete spawning in *A. japonicus* [31]. Furthermore, NGIWYamide is the prototype for a family of neuropeptides in deuterostomian invertebrates that have a characteristic Asn-Gly (NG) motif and which are therefore known as “NG peptides” [32]. Using a hypothetical partial precursor sequence for NGIWYamide (KRNGIWYGKRNGIWYGKRNGIWYGKR) as a query sequence in a BLAST search of sequence data from the sea urchin *S. purpuratus*, a protein containing two copies of a putative neuropeptide with the sequence NGFFFamide was identified [33]. Analysis of the sequence of the NGFFFamide precursor protein revealed the presence of an N-terminal signal peptide, indicating that NGFFFamide is a secreted neuropeptide in sea urchins. Furthermore, analysis of the *in vitro* pharmacological effects of NGFFFamide revealed that it causes contraction of sea urchin muscle preparations, consistent with the muscle-contracting action of NGIWYamide in *A. japonicus*. Interestingly, analysis of the sequence of the NGFFFamide precursor revealed the presence of a C-terminal neurophysin-like domain. Neurophysins are polypeptides that hitherto were thought to be uniquely associated with the precursors of vasopressin/oxytocin-type neuropeptides and which are required for biosynthesis of these neuropeptides [34], [35]. Therefore, the discovery that the sea urchin NGFFFamide precursor also has a neurophysin domain was a surprising finding that invited explanation, and it was postulated that the NGFFFamide precursor may have originated as consequence of complete or partial duplication of a gene encoding a vasopressin/oxytocin-type neuropeptide precursor [33]. Subsequently, it was discovered that genes encoding NG peptide type precursors with a neurophysin domain are not unique to sea urchins but are also present in other deuterostomian invertebrates [32]. Thus, a gene in the hemichordate *Saccoglossus kowalevskii* encodes a protein with a C-terminal neurophysin domain and five copies of the putative neuropeptide NGFWNamide and one copy of NGFYNamide, whereas in the cephalochordate *Branchiostoma floridae* there is a NG peptide precursor with a C-terminal neurophysin domain and two copies of the putative neuropeptide SFRNGVamide. Discovery of these genes demonstrated that the origin of neurophysin-containing NG peptide precursors could be traced back at least as far as the common ancestor of the deuterostomes. Therefore, it was anticipated that the precursor of NGIWYamide, the prototype of the NG peptide family, would also have a neurophysin domain. However, the identification of the NGIWYamide precursor in *A. japonicus*, as reported here, has revealed that it contains five copies of NGIWYamide but lacks a C-terminal neurophysin domain. Therefore, presumably the neurophysin domain has been lost from NG peptide precursors in the sea cucumber lineage that gave rise to *A. japonicus*.

It is noteworthy that the *B. floridae* NG peptide SFRNGVamide is identical to the N-terminal region of a vertebrate neuropeptide named “neuropeptide S” or NPS [32], [36]. This high level of sequence similarity suggests that NPS may in fact be a vertebrate representative of the NG peptide family in deuterostomes. However, unlike the SFRNGVamide precursor in *B. floridae*, the NPS precursor does not have a C-terminal neurophysin domain [36]. Therefore, NPS precursors in vertebrates and the NGIWYamide precursor in *A. japonicus* may be examples of NG peptide precursors that have lost their neurophysin domain. This suggests that the neurophysin domain is dispensable and may not be essential for NG peptide biosynthesis. However, if this is the case then why has it been retained in other lineages? Experimental studies that investigate the role of neurophysin in NG peptide biosynthesis are now required to address this issue.

### Identification of the stichopin precursor provides further evidence that stichopin is a secreted peptide signalling molecule in *A. japonicus*

The peptide stichopin (DRQGWPACYDSKGNYLC) was originally isolated from *A. japonicus* on account of its inhibitory modulating effect on electrically induced contractions of radial longitudinal muscle (RLM) preparations [22], [23]. Subsequently, it was found that stichopin also inhibits acetylcholine induced stiffening of the collagenous body wall of *A. japonicus* [7]. Collectively, these data indicated that stichopin may act physiologically as a signalling molecule that regulates the mechanical properties of body wall muscle and connective tissue. However, because stichopin was originally isolated from whole body wall extracts, it was not known which tissue(s) and cell types stichopin is derived from. To address this issue, antibodies to stichopin were generated and used for immunocytochemical studies on *A. japonicus* [25]. Interestingly, stichopin-immunoreactivity (ir) was found exclusively in connective tissue associated with a variety of different organ systems. At a cellular level stichopin is expressed in two types of cells: firstly, putative neuronal cells with processes and secondly, oval shaped cells without processes (non-neuronal). Furthermore, electron microscopic studies revealed that the stichopin-immunoreactive oval shaped cells have characteristics of secretory cells. Based on these data, it was proposed that stichopin acts as a neuropeptide-type signalling molecule (derived from neurons) and as a hormone (derived from oval-shaped secretory cells), with roles specifically associated with regulation of connective tissue [25].

The discovery of the 39 amino acid residue stichopin precursor, as reported here, supports the hypothesis that stichopin acts as a neuropeptide-type signalling molecule and as a peptide hormone in sea cucumbers. In particular, the presence of an N-terminal signal peptide is consistent with targeting of stichopin to the regulated secretory pathway. A noteworthy feature of the stichopin precursor is that it consists solely of a signal peptide (residues 1–22) followed by the stichopin peptide (residues 23–39), which is the simplest type of precursor protein for neuropeptides and peptide hormones. Furthermore, determination of the sequence of the stichopin precursor has revealed polymorphisms in the sequence of stichopin at residues 12 and 16, which may reflect differences between the population of *A. stichopus* that was used for peptide purification and sequencing [22] and the population that was used for transcriptome sequencing [10].

### Identification of the GN-19 precursor protein indicates that GN-19 is a secreted peptide signalling molecule in *A. japonicus*

The peptide GN-19 (GGRLPNYAGPPRMPWLIHN) was originally identified as peptide derived from *A. japonicus* body wall extracts that causes contraction or relaxation of intestine preparations from this species, but has no effect on the radial longitudinal muscle [23]. Here the GN-19 precursor has been identified, revealing that GN-19 is derived from an 82 residue protein with a predicted N-terminal signal peptide and with the GN-19 sequence bounded by putative dibasic cleavage sites. Importantly, these features indicate that GN-19 is a secreted signalling molecule. The identification of a cDNA encoding GN-19 will facilitate analysis of the expression of this peptide in *A. japonicus* and investigation of its physiological roles as a putative neuropeptide or endocrine-type signalling molecule.

### Identification of the GLRFA precursor protein indicates that GLRFA is a secreted signalling molecule in *A. japonicus*

The peptide GLRFA was originally identified as a peptide derived from *A. japonicus* body wall extracts that causes contraction of intestine preparations from this species and that potentiates electrically induced contraction of radial longitudinal muscle preparations [22], [23]. Here the GLRFA precursor has been identified, revealing that GLRFA is derived from a 62 residue protein with a predicted N-terminal signal peptide and with a single copy of the GLRFA sequence bounded N-terminally by a putative dibasic cleavage site and bounded C-terminally by a putative monobasic cleavage site. As with the GN-19 precursor, these features indicate that GLRFA is a secreted signalling molecule and identification of cell types that express the GLRFA precursor in *A. japonicus* will facilitate investigation of its physiological roles as a putative neuropeptide or endocrine-type signalling molecule.

### Holokinins: collagen-derived bioactive peptides?

Four holokinins have been isolated from *A. japonicus*: holokinin-1 (PLGYMFR) and derivatives of holokinin-1 with either an oxidised methionine residue (PLGYM(O)FR, holokinin-2) or with a brominated tyrosine residue (PLGY(Br)MFR, holokinin-3) or with N-terminal truncation (GYMFR, holokinin-1(3–7)). All four peptides cause inhibition of electrically induced contraction of radial longitudinal muscle (RLM) preparations and contraction of intestine preparations [22], [23]. Furthermore, it has been discovered that holokinin-1 and holokinin-2 also cause softening of the collagenous body wall dermis of *A. japonicus* [7]. But nothing is known about how holokinins exert effects on muscle or connective tissue. It has been noted, however [22], that holokinins share structural similarity with bradykinin (RPPGFSPFR), a vasoactive peptide in mammals that is derived from the precursor protein kininogen through the action of kallikrein serine proteases [37]. Bradykinin exerts effects by binding to the G-protein coupled receptors B1 and B2 [37]. Likewise, holokinins may exert effects on muscle contractility in sea cucumbers by binding to receptors that are expressed pre-synaptically on motor nerves or that are expressed post-synaptically by muscle cells. Similarly, the softening action of holokinins on collagenous body wall preparations [7] might be mediated by receptors expressed by cells located in connective tissue. However, potential alternative mechanisms of action for these peptides can be envisaged with the discovery, as reported here, that holokinins may be derived from proteolysis of an alpha-5 type collagen in *A. stichopus*.

Mutable connective tissue in echinoderms changes between stiff and pliant states and the collagenous body wall of sea cucumbers is one of the best-studied examples of this [6]. Importantly, changes in the stiffness of the body wall dermis are thought to be controlled by the nervous system [6] and evidence in support of this has been provided by immunocytochemical studies revealing that neuropeptides that affect the stiffness of the body wall *in vitro* (e.g. NGIWYamide and stichopin) are present in nerve process and/or endocrine-type cells located within the collagenous dermis of the body wall [24], [25]. It is postulated that neural control of the mechanical state of this mutable connective tissue is mediated by “juxtaligamental cells” located amongst the collagen fibrils. Thus, neurotransmitters or neuropeptides released by nerves are thought to stimulate juxtaligamental cells to release proteins that affect interaction between collagen fibrils [6]. For example, a “stiffening” protein that binds to collagen fibrils via surface glycosaminoglycans (GAGs) has been isolated and sequenced and is known as tensilin [38]. Furthermore, it is proposed that release of a tensilin protease by juxtaligamental cells cleaves tensilin near its GAG-binding site and this causes softening by allowing collagen fibrils to slide past each other [6]. The discovery that holokinins, peptides that cause softening of the body wall dermis *in vitro*, may be derived from proteolysis of an alpha-5 type collagen in *A. stichopus* suggests other mechanisms by which the stiffness of dermis may be regulated. Whilst the possibility remains that the softening effect of holokinins *in vitro* is a pharmacological phenomenon that has no relevance to physiological control mechanisms, if proteolytic generation of holokinins from alpha-5 type collagen does occur *in vivo* then potential mechanisms by which these peptides exert their effects physiologically can be envisaged. For example, holokinins might cause softening of mutable connective tissue in the body wall dermis by competitively blocking interaction between the PLGYMFR sequence located in fibrils that contain alpha-5 type collagen and putative PLGYMFR-binding sites on other adjacent fibrils. Interestingly, previous studies on the endoskeleton of the sea urchin *Paracentrotus lividus* have revealed that in this collagenous tissue alpha-5 type collagen chains form heteromeric molecules with two alpha-1 type collagen chains. Furthermore, alpha-5 N-terminal propeptides are found at the surface of collagen fibrils and it has been suggested that these proteins may link collagen fibrils and therefore “might be of importance in the unique properties of mutable collagenous tissues” [39]. Thus, the discovery that holokinins, peptides that cause softening of body wall collagenous tissue in sea cucumbers, appear to be derived from alpha-5 type collagen is intriguing. Collectively, these findings provide a strong basis for further investigation of the roles of alpha-5 type collagen in mechanisms of mutable connective tissue in echinoderms.

### Other myoactive peptides derived from *A. japonicus*

For six of the myoactive peptides isolated from *A. japonicus* (KHKTAYTGIamide, APHAIRPPSG, GYWKDLDNYVKAHKT, MPMNPADYFSRGTVYIPTRDS, GPSANSFTYGRCADDRC and KGKQKGAGRGKGN) no putative precursor proteins were identified based on BLAST analysis of the *A. japonicus* transcriptome sequence data, which may indicate that the precursors of these peptides are encoded by low abundance transcripts or that mRNAs encoding precursors of these peptides are not expressed in the tissues/organs that were used for production of cDNA libraries for sequencing [10].

A putative precursor for the myoactive peptide GYI was identified as the sea cucumber homolog of human UFM1-specific ligase 1, but the physiological relevance of this is uncertain. The GYI sequence in *A. japonicus* UFM1-specific ligase 1 is bounded by basic amino acid residues that could serve as substrates for endopeptidases. However, it is possible that the proteolysis of *A. japonicus* UFM1-specific ligase 1 that putatively gives rise to the GYI peptide is simply an artifact of peptide extraction *in vitro* and not a physiological phenomenon.

Analysis of the transciptome data for putative precursors of the three *Apostichopus* SWYG peptides revealed a potential precursor for SWYG-3 but not for SWYG-1 and SWYG-2. However, analysis of the sequence of the protein containing the sequence of SWYG-3 (SWYGSLA) indicates that it may in fact be derived from a bacterial species that is associated with *A. japonicus.*

### Conclusions

The GYI and SWYG-3 peptides highlight the difficulties associated with using extracts of organs or other body parts for discovery of putative peptide signalling molecules. Some of the peptides identified as bioactive molecules biochemically may be generated artifactually as a consequence of *in vitro* proteolysis of proteins derived from the tissues/organs analysed or from commensal/symbiotic bacterial species associated with the study organism. The utilization of transcriptome sequence data, as employed here, is a valuable complementary approach in discovery of peptide signalling molecules because by identifying the precursor proteins of bioactive peptides it is possible to determine which peptides are likely to be utilized as signalling molecules *in vivo*. Here precursor proteins for SALMFamide neuropeptides, NGIWYamide, stichopin, GN-19 and GLRFA have been identified in the *A. japonicus*. For SALMFamides, NGIWYamide and stichopin, there was already evidence from immunocytochemical studies that these peptides act as secreted neuropeptide or endocrine-type signalling molecules [20], [24], [25], and the identification of the precursors of these peptides has provided additional evidence for this. Furthermore, in the case of SALMFamide precursor it has revealed the existence of novel peptides. The identification of the precursor proteins for GN-19 and GLRFA is important because it provides a strong rationale for further investigation of these particular peptides as neuropeptide or endocrine-type signalling molecules in *A. japonicus*. Lastly, whilst it appears that the holokinins may not act as conventional neuronal or endocrine-type secreted peptide signalling molecules in *A. japonicus*, the discovery that these peptides may be derived from collagen proteolysis has provided a potentially important new insight on the physiological mechanisms that mediate regulation of the mechanical properties of connective tissue and muscle in echinoderms.

## Supporting Information

Figure S1 **Putative protein from which the peptide SWGSLA is derived.**
(PDF)

Figure S2 **Putative protein from which the peptide GYI is derived.**
(PDF)
