# Supplementary material for: The Protein Precursors of Peptides That Affect the Mechanics of Connective Tissue and/or Muscle in the Echinoderm Apostichopus japonicus
Source: PLoS One. 2012 Aug 31;7(8):e44492. doi: 10.1371/journal.pone.0044492 (PMC3432112; doi:10.1371/journal.pone.0044492)
Supplement: Figure S1 — Putative protein from which the peptide SWGSLA is derived. (PDF) [file pone.0044492.s001.pdf]

```
1                                                                    c
2   tcggtcaaaactttcgccgcttccatcgaggacgaaattcttgacgcggatgcccttagt
     S   V   K   T   F   A   A   S   I   E   D   E   I   L   D   A   D   A   L   S    20
62  cgccgcggcggatggtttcatagactgttccatcatcaccaccatggtgcaccagccgac
     R   R   G   G   W   F   H   R   L   F   H   H   H   H   H   G   A   P   A   D    40
122 aacaagaaccagccaaaacagagagaggaaagacaactcatacatgataacagagagaat
     N   K   N   Q   P   K   Q   R   E   E   R   Q   L   I   H   D   N   R   E   N    60
182 gccatgctagatgacttcgagaagaaaatggctgagttatctccagcggagcgttttgac
     A   M   L   D   D   F   E   K   K   M   A   E   L   S   P   A   E   R   F   D    80
242 tggcatgctctcttacacagtgtcagtaaacatgttgctaaacacgttgcagagaactta
     W   H   A   L   L   H   S   V   S   K   H   V   A   K   H   V   A   E   N   L   100
302 aaagcgagagtgttggccgaagaaggggagcgactgcccgagggatacgacgagctaaca
     K   A   R   V   L   A   E   E   G   E   R   L   P   E   G   Y   D   E   L   T   120
362 cctgaagagagaagctggtatggctccctggcaagtgcactccttggtagatgaatactg
     P   E   E   R   S   W   Y   G   S   L   A   S   A   L   L   G   R   *           137
422 cgggaaagaagcttgtatctgacccaaactgaccgatatttagtctatagacacccatcc
482 tatcaagtgaaatatttcccttgtactttagtattgtttcgatttcaagtaatggtggtg
542 gattgactttttggaataaaaatcagaaaataaagtaagcacgatct
```

Figure S1.
Putative protein from which the peptide SWGSLA is derived. The sequence of isotig 11737 is shown (lowercase, 588 bases) with the partial sequence of the protein that it encodes shown underneath in bold uppercase (137 amino acid residues). The SWGSLA sequence is shown in red and the asterisk shows the position of the stop codon. Submission of this protein sequence as query in a BLAST search of the GenBank protein database reveals that it shares sequence similarity with proteins of bacterial origin (see text in results section).
