# Supplementary material for: The Protein Precursors of Peptides That Affect the Mechanics of Connective Tissue and/or Muscle in the Echinoderm Apostichopus japonicus
Source: PLoS One. 2012 Aug 31;7(8):e44492. doi: 10.1371/journal.pone.0044492 (PMC3432112; doi:10.1371/journal.pone.0044492)
Supplement: Figure S2 — Putative protein from which the peptide GYI is derived. (PDF) [file pone.0044492.s002.pdf]

```
1    gaccacaaacttgtcaacatggccagcagtgactgggaagaagtgaaacggcttgcggca
      D  H  K  L  V  N  M  A  S  S  D  W  E  E  V  K  R  L  A  A   20
61   gatttcaagcgagcacagttaacgtcgtcatcacagagattatcagaaagaaactgtgtg
      D  F  K  R  A  Q  L  T  S  S  S  Q  R  L  S  E  R  N  C  V   40
121  gagatactatccaaactcattgaagaaaaacaaattgaagtcatttactcactagacggc
      E  I  L  S  K  L  I  E  E  K  Q  I  E  V  I  Y  S  L  D  G   60
181  aaagaatatgtcacaccttcccagctatttaaagaaatacgagatgaacttatagtacat
      K  E  Y  V  T  P  S  Q  L  F  K  E  I  R  D  E  L  I  V  H   80
241  ggaggaagagtaaacttggtagatcttcaacaaaccattgggatcgagctctcccaaatc
      G  G  R  V  N  L  V  D  L  Q  Q  T  I  G  I  E  L  S  Q  I  100
301  gaaaccaaggctgccgagatcgtcaggtccgaccagtccgtttcgttagtgttaggacag
      E  T  K  A  A  E  I  V  R  S  D  Q  S  V  S  L  V  L  G  Q  120
361  cttatagacgattcttacctggatcatctggccgaagaaataaacgaacagcttcaagag
      L  I  D  D  S  Y  L  D  H  L  A  E  E  I  N  E  Q  L  Q  E  140
421  aacggccaagttactattccagcacttacccagtcgctcgatcttcctgccgacttttta
      N  G  Q  V  T  I  P  A  L  T  Q  S  L  D  L  P  A  D  F  L  160
481  tcagaggctattgaatcacgtattggtcggttaatcaatggggagatcgatccatacgac
      S  E  A  I  E  S  R  I  G  R  L  I  N  G  E  I  D  P  Y  D  180
541  agggacgtcatcttcacccaggcctttgtcgagaggaatacagcaaaggtcagaggtgtc
      R  D  V  I  F  T  Q  A  F  V  E  R  N  T  A  K  V  R  G  V  200
601  ttcagtgctataactaggccaacatctgtacaaagtatcttgaacaagtacaaattccct
      F  S  A  I  T  R  P  T  S  V  Q  S  I  L  N  K  Y  K  F  P  220
661  gaaaaactcttttatagtgtcttagaaaagcttgtcaactctggtcgcttgaatggtgac
      E  K  L  F  Y  S  V  L  E  K  L  V  N  S  G  R  L  N  G  D  240
721  atcatcggtggccgtcaagacaaagcttcttacgtcccagagatattttctaaaacgcag
      I  I  G  G  R  Q  D  K  A  S  Y  V  P  E  I  F  S  K  T  Q  260
801  aataactgggtcgattctttctacaaacaaaatggatacttggaatatgatacgttgtca
      N  N  W  V  D  S  F  Y  K  Q  N  G  Y  L  E  Y  D  T  L  S  280
861  agacttggggtgacagaccctaaaggttacatcaagaggcggttcaaggcggagaaattc
      R  L  G  V  T  D  P  K  G  Y  I  K  R  R  F  K  A  E  K  F  300
921  atccacctgaaggctgcatccatcgggaagatactgcaggaccaagtagatgctaccatc
      I  H  L  K  A  A  S  I  G  K  I  L  Q  D  Q  V  D  A  T  I  320
981  gatgaagctgtgagaacatcaagttgggtggatgtcatgcctatcctgccatcgtccctc
      D  E  A  V  R  T  S  S  W  V  D  V  M  P  I  L  P  S  S  L  340
1041 tccagacaagacgttaaccaactcattcaagatatttttgaaaccgaagagtaaactcggg
      S  R  Q  D  V  N  Q  L  I  Q  D  I  L  K  P  K  S  K  L  G  360
1101 attaaggtcttcaacgacaccatcatctgcagcaaagccttcctcgacgagtgcttgacg
      I  K  V  F  N  D  T  I  I  C  S  K  A  F  L  D  E  C  L  T  380
1161 atctttgaaccggtcatgaaggagaaggctcaaaaggatgctgtgactcatcgcgacttt
      I  F  E  P  V  M  K  E  K  A  Q  K  D  A  V  T  H  R  D  F  400
1221 ttcctacacaaagataagaaagcggtggcagcattcagcggctcggacggaaaacaagac
      F  L  H  K  D  K  K  A  V  A  A  F  S  G  S  D  G  K  Q  D  420
1281 aagaaggatgagagaaggaaaaaagctgcaggtggcggtggcagcagtgggaaatccggc
      K  K  D  E  R  R  K  K  A  A  G  G  G  G  S  S  G  K  S  G  440
1341 gttgccagcacaagtcgagaggtcaagactaagaaggtcaagaagggccggaacaaagac
      V  A  S  T  S  R  E  V  K  T  K  K  V  K  K  G  R  N  K  D  460
1401 ccggtagaaccgttagaagaagaatcgtcgacgagcagcagaggtcaaagccaggagttg
      P  V  E  P  L  E  E  E  S  S  T  S  S  R  G  Q  S  Q  E  L  480
1461 gagttctatccggtggagaaaatagagggaatattaaagaccaaactacgggattgccca
      E  F  Y  P  V  E  K  I  E  G  I  L  K  T  K  L  R  D  C  P  500
1521 gaagagttgcacacagaattagcggaggatttattaagacctttgaccagacgatatcaa
      E  E  L  H  T  E  L  A  E  D  L  L  R  P  L  T  R  R  Y  Q  520
1581 gaaacagccaaggaggcttacatggcgttagcaggggggttcctctgctgcagagaggaga
      E  T  A  K  E  A  Y  M  A  L  A  G  G  S  S  A  A  E  R  R  540
1641 aagacccacgagaatgtccaggagaaggtcaactcactctggaccaacgtcaaactcttt
      K  T  H  E  N  V  Q  E  K  V  N  S  L  W  T  N  V  K  L  F  560
1681 gagaaggggattaatcaatttacagatgaagatgtggtcagccaactgaacaagtacc
      E  K  G  I  N  Q  F  T  D  E  D  V  V  S  Q  L  N  K  Y     579
```

Figure S2.
Putative protein from which the peptide GYI is derived. The sequence of isotig 09011 is shown (lowercase, 1738 bases) with the partial sequence of the protein that it encodes shown underneath in bold uppercase (579 amino acid residues). The GYI sequence is shown in red. Submission of this protein sequence as a query in a BLAST search of the GenBank protein database indicates that it is an *A. stichopus* homolog of human UFM1-specific ligase 1 (see text in results section).
